# Multidimensional Transcriptomics Reveals the Key Genes and Pathways Regulating the Acidity of Apples

**DOI:** 10.3390/cimb47050341

**Published:** 2025-05-08

**Authors:** Wenyuan Yang, Hang Yu, Lian Tao, Hongjiang Xie

**Affiliations:** 1 Horticulture Research Institute, Sichuan Academy of Agricultural Sciences, Chengdu 610066, China; yangwy0215@scsaas.cn (W.Y.); yuh0928@scsaas.cn (H.Y.); tao_lian@scsaas.cn (L.T.); 2 Key Laboratory of Horticultural Crops Biology and Germplasm Enhancement in Southwest, Ministry of Agriculture, Chengdu 610066, China

**Keywords:** apple, Western Sichuan plateau, mutant variety, fruit ripening, acidity

## Abstract

Low-acid apples are popular among consumers, but the mechanisms behind the complex differences in acidity among varieties that are caused by high altitude are not clear. In this study, we used the ‘Golden Delicious’ apple and its superior variant in the Western Sichuan Plateau of China to analyze organic acid composition, content, and the expression levels of related regulated genes during fruit development. We found that the organic acid content in the variant was significantly lower than that in the ‘Golden Delicious’ apple. In both apples, quinic and malic acids were the predominant organic acids, while citric and tartaric acids were present in lower amounts. In this multidimensional regulatory study, we used transcriptome sequencing, cluster analysis, and weighted gene co-expression network analysis (WGCNA) to reveal that differentially expressed genes are enriched in multiple pathways affecting fruit acidity during apple development; malate dehydrogenase (MDH) affects the malic acid content of fruits of different varieties; and H^+^-ATPase (VHA) mainly regulates the content of vacuolar organic acids, which affects fruit acidity. Additionally, we performed qRT-PCR experiments to validate our results. This study provides molecular insights into the mechanisms by which low-acidity traits form in apples and offers a theoretical basis for regulating the flavor of fleshy fruits.

## 1. Introduction

Fruit flavor is primarily determined by the composition of organic acids; soluble sugars; specific secondary metabolites, such as flavonoids and phenolic compounds; and volatile organic compounds, many of which also contribute to a fruit’s nutritional value and health-promoting properties [1,2]. Organic acids, such as malic, citric, and tartaric acids, along with soluble sugars like sucrose, fructose, and glucose, are the key contributors to the sour and sweet flavors of ripe fruits [3]. The specific types and concentrations of these compounds directly influence fruit taste, as different organic acids and sugars vary significantly in acidity and sweetness [4,5]. Furthermore, organic acids play a vital role in fruit coloration during ripening, extending the shelf life of fresh produce, and supporting fruit reprocessing [6]. Consequently, optimizing the composition of organic acids and soluble sugars has become a central objective in global fruit breeding programs.

The apple (*Malus domestica* Borkh.) is a fruit of significant economic importance in temperate regions and is widely consumed worldwide due to its unique flavor and health benefits [7]. Morphological and genetic evidence indicates that cultivated apples were originally domesticated from the wild apple (*Malus sieversii*), which is native to the Tian Shan Mountains in Central Asia [8,9]. As apples spread along the Silk Road from Asia to Europe, their genetic makeup evolved through hybridization with local wild apple species. Many current studies focus on increasing the level of specific compounds in Golden Delicious apples to improve their flavor and color. For example, UV-B/C have been used to significantly enhance anthocyanin accumulation, resulting in a 90% increase in color development. However, some experiments have confirmed that such treated apples may be harmful to the health of rats [10]. Another technique for improving various qualities of apples is natural genetic hybridization, which can be used to improve size, color, acidity, and sweetness [11]. During this process, human selection favored traits such as lower acidity, as wild apples are typically characterized by high organic acid and tannin content, resulting in an unpleasant sourness and astringency. Lower acidity not only enhances the flavor of apples but also benefits individuals that are sensitive to fruit acids [12]. Consequently, reducing acidity has become a key focus in the functional breeding of apple varieties.

A variety of molecular mechanisms have been confirmed to affect fruit acidity in fleshy fruits. The production of oxaloacetate (OAA) from phosphoenolpyruvate (PEP) in mitochondria in the presence of phosphoenolpyruvate carboxylase (PEPC) and phosphoenolpyruvate carboxykinase (PEPCK) can be used directly for the synthesis of citric acid. Citrate synthase (CS) uses OAA in the formation of citrate, the intermediate that starts the TCA cycle malate dehydrogenase (MDH) determines the amount of malic acid in the cell during this cycle [13,14]. Mitochondrial MDHs play a role in the TCA cycle, whereas cytoplasmic MDHs are involved in both acid metabolism within plant tissues and carbon dioxide fixation in C4 plants [14,15]. An increase in PEPC, MDH, and CS in tomatoes will increase the total amount of organic acids in the fruits [16]. This phenomenon has also been observed in peaches [17]. The transport and storage of organic acids into vacuoles are complex processes involving numerous transporters, proton pumps, and enzymes [18]. Tt has been found that there are two unique proton pumps in the vacuole membrane, H^+^-ATPase and H^+^-PPase, which constantly transport protons from the cytoplasm to the vacuole and play an important role in the storage of organic acids in the vacuole [19]. The mechanism of acidity regulation via vacuolar P-ATPases (PH1 and PH5) has been confirmed by numerous studies in lemon [20], sweet orange [21], and grapefruit [22]. There have been many studies on the mechanisms of acidity regulation in apples, such as promoter variations of malate dehydrogenase1 (MDH1) gene affect MdbHLH74 binding, reducing MDH1 transcription and apple malic acid levels,, and WRKY126 can also alter malic acid accumulation in malic acidic fruits by regulating the level of malate dehydrogenase [23,24]. Malic acid is transported to the vacuoles s with the help of a proton pump, thus affecting fruit acidity [25]. However, little research has been conducted on the acidity of varietal low-acid varieties of apples. Therefore, studying the composition of and metabolic changes in organic acids in low-acid apple varieties during different ripening stages is particularly important for breeding efforts and enhancing fruit quality.

China is the world’s leading producer of apples [26], with a cultivation area of 2 million hectares and an annual production exceeding 40 million tons, accounting for over 50% of global apple acreage and output. In China, the high-altitude, temperature-differentiated southwestern plateau region is an important apple-producing area with unique growing conditions that contribute to varietal variation. Among the many widely grown apple varieties, Golden Delicious (GD) is a distinctive apple variety that is renowned for its glossy surface, brilliant color, elongated shape, fragrance, flavor, and excellent texture. The Western Sichuan Plateau’s high temperature differentials and high-altitude (1400–2700 m), high-light, and low-oxygen environments influence many traits of apples, including good qualities, such as better flavor and texture, and some negative ones, such as skin that is more susceptible to injury. A low-acidity mutation variant (GDM) of the GD apple was identified during production practices, thus providing valuable material for studying the mechanisms underlying low-acidity traits in apples. In this study, we investigated the multidimensional transcriptomics of GD and GDM apples and identified the key genes and biological pathways that regulate fruit acidity. We hypothesize that the changes in apple acidity at high altitudes are due to significant changes in the expression levels of critical genes that are differentiated from those in apples at low altitudes. This provides a theoretical basis for the future breeding of low-acidity and high-quality apple varieties through molecular breeding techniques, as well as a foundation for apple flavor improvement and genetically improving other fruits at high altitudes.

Our research pioneers the exploration of fruit acidity regulation mechanisms in apples grown in high-altitude regions. The unique environmental conditions of high-altitude areas—such as hypoxia, intense UV radiation, and significant diurnal temperature fluctuations—have endowed apples with novel phenotypic traits. This study identified distinctive acidity-regulating genes in apple cultivars grown in high-altitude conditions, thus providing critical targets for marker-assisted breeding. We believe these findings will deepen the understanding of organic acid regulation in apples and establish a robust theoretical foundation and technical framework for breeding fleshy fruit varieties adapted to other high-altitude regions.

## 2. Materials and Methods

### 2.1. Plant Materials and Growth Conditions

The ‘Golden Delicious’ apple and the mutant variant termed GDM were collected from the apple experimental orchard in Aba Tibetan and Qiang Autonomous Prefecture (31.14° N, 102.37° E, Elevation 2450 m), Sichuan Province, China, with an age of 8 years, a rootstock of Begonia minor, a plant spacing of 3 × 4 m, an area of 667 m^2^, a medium level of cultivation and management, and basically the same growth potential.

### 2.2. Sampling Method

In this study, three GD apple trees with uniform growth and GDM apple trees were selected and sampled at 27 d, 47 d, 67 d, and 147 d after flowering, until the fruits reached physiological maturity. (The sampling time was determined based on a pre-test of the fruit development dynamics of apples in the Western Sichuan Plateau. Of these sampling times, 27 d, 47 d, and 67 d fall into the fruit expansion stage and 147 d into the fruit ripening stage.) Fresh fruits were peeled, cored, and chopped; quickly frozen and mixed with liquid nitrogen; and then put into an ultra-low-temperature refrigerator (−80 °C) in two groups for backup. Three trees were observed for their apple formation for 3 consecutive years (2018–2020), an average value was taken.

### 2.3. Extraction of Acid Components and Determination

A total of 2 g of fresh flesh of apple samples was accurately weighed and placed into a mortar, and 5 mL of ultrapure water was added for grinding and mixing. Ultrasonication was carried out at room temperature for 20 min, at 4 °C, followed by 12,000 *g* centrifugation for 15 min. The supernatant was then transferred to a 10 mL volumetric flask, and 5 mL of ultrapure water was added to the residue and centrifuged again. The supernatant was combined twice and fixed to 10 mL and then filtered via a 0.45 μm microporous filter membrane. The filtrate was then filtered through a 0.45 μm microporous membrane and placed in a 1.5 mL injection bottle. High-performance liquid chromatography (HPLC–Agilent 6400) was used for the determination of acid fractions [27]. The chromatographic conditions for the determination of organic acids were as follows: a Shim-pack VP-ODS C_18_ column was used at 25 degrees Celsius; mobile phase A was 0.1% metaphosphoric acid; mobile phase B was 3% methanol; the total flow rate was 0.5 mL/min; the detection wavelength was 210 nm; a Shimadzu SPD-10A ultraviolet detector was used; and the injection volume was 20 μL. The content of each organic acid was calculated according to the peak area of the sample and the standard curve of each organic acid.

### 2.4. RNA Extraction and Library Construction

We selected 27 d, 47 d, 67 d, and 147 d as developmental stages 1, 2, 3, and 4 for RNA-seq. The first three of these sampling times fell into the expansionstage, and the fourth fell into the maturation stage. The total RNA of the samples was extracted using RNAprep Pure Polysaccharide Polyphenol Plant Total RNA Extraction Kit (DP441) from Tiangen Biochemical Technology (Beijing, China) Co. RNA integrity and DNA contamination were assessed via agarose gel electrophoresis; RNA concentration was quantified via Nanodrop and detected using an ND-1000 spectrophotometer; and RNA integrity was evaluated via Agilent 2100. According to the relevant operating instructions of Illumina, we prepared the samples using the Illumina Tru Seq™ RNA Sample Preparation Kit. The constructed libraries were sequenced with Illumina HiSeqTM 2500 (Illumina, San Diego, CA, USA).

### 2.5. Transcriptomic Analysis

All the raw sequencing data were quality-controlled using FASTP v0.24.0 [28] to obtain high-quality clean data with adapters removed. HISAT2 v2.2.1 [29] was used to map to the reference genome [8]. All gene expression levels were evaluated using the software StringTie v1.3.3 [30]. A Limma v3.62.2 (Moderated F-test) package based on R v4.4.2 was used to calculate the statistics of differentially expressed genes (Log_2_|FC|  >  2 and FDR  <  0.05) [31]. The Mfuzz v2.66.0 method was used in this study for gene clustering [32]. A weighted correlation network analysis was performed based on the R package WGCNA v1.73 [33]. For a functional enrichment analysis of key genes, we used GOATOOLS v1.4.12 software (find_enrichment.py −pval = 0.05 −indent −outfile) [34].

### 2.6. qRT-PCR Verification

Total RNA for fluorescence quantitative analysis was extracted using an Invitrogen Trizol RNA isolation kit and reverse-transcribed into cDNA using a TaKaRa PrimeScriptTM 1st stand cDNA Synthesis Kit reverse transcription kit. Actin was selected as the internal reference gene, and from the differentially expressed genes, six differentially expressed up-regulated genes and three differentially expressed down-regulated genes were randomly selected for qRT-PCR verification. These differentially expressed genes and primer sequences are shown in Table A1. Three biological replicates were performed for each reaction, and the reaction volume was 10 μL. The validation data were calculated and analyzed using the 2^−△△Ct^ method [35].

## 3. Results

### 3.1. Organic Acid Profiles of GD and GDM Apples During Development

**Figure 1:**
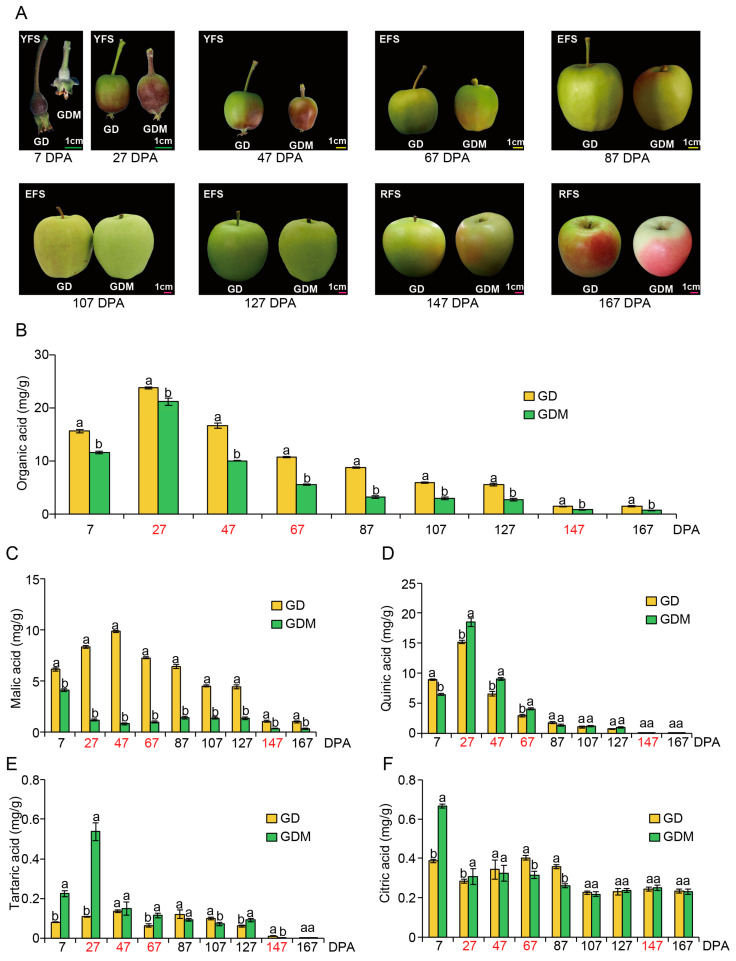
The features of Golden Delicious (GD) and Golden Delicious Mutation (GDM) apples observed during fruit ripening. (**A**) Changes in the appearance of apple fruit during development. The line segment in this figure is a scale of 1 cm. YFS, EFS, and RFS represent young fruit stage, expansion fruit stage, ripening fruit stage, respectively. (**B**–**F**) Changes in total organic (**B**), malic (**C**), quinic (**D**), tartaric (**E**), and citric acid (**F**) content in 10 replicates during fruit ripening. The red font represents the stage in which RNA-seq was performed. DPA: Days Post-Anthesis. Groups labeled with the same letter (a, a) are not significantly different, while those with different letters (a, b) indicate statistically significant differences.

In this study, nine stages (7, 27, 47, 67, 87, 107, 127, 147, and 167 Days Post-Anthesis, DPA) of GD and GDM apples were selected for phenotypic observation and organic acid determination. Regarding fruit size, GD exhibited larger dimensions than GDM apples during the early developmental stage, with both gradually converging in later phases. In terms of fruit coloration, both apple varieties demonstrated a transition from red to green during growth, followed by color development at maturity—particularly pronounced in GDM apples—indicating that plateau environments promote pigmentation in green apples. Additionally, GDM fruits exhibited a smoother surface texture, whereas GD fruits displayed numerous lenticels (fruit spots) and russeting (surface roughness) (Figure 1A). Most notably, GD had a more acidic taste compared to GDM apples.

To investigate the underlying causes of the acidity difference between the two apple varieties, we conducted quantitative analyses of various organic acid contents in the fruits. For total organic acids, both GD and GDM apples showed elevated levels between 7 and 27 DPA, followed by a gradual decrease in the subsequent stages. At each time point, the organic acid content of GD was consistently higher than that of GDM apples, indicating that GDM apples are a high-quality variety with low-acid characteristics (Figure 1B). As for the composition of organic acids, malic, citric, quinic, and tartaric acids were the main components [36]. Therefore, we also determined the content of these acids separately. We found that quinic and malic acids were the predominant components of the total organic acids in both GD and GDM apples, while tartaric and citric acids comprised smaller proportions. Regarding malic acid, GD consistently exhibited higher levels than GDM apples. The peak malic acid levels in GD occurred at 47 DPA and then continued to decline, whereas the organic acid levels in GDM apples decreased after 7 DPA, slightly increased in later stages, and then decreased again at 147 DPA (Figure 1C). For quinic acid, both GD and GDM apples showed trends consistent with the total organic acid pattern, indicating that quinic acid played a major role in determining the total organic acid content in both varieties. Notably, GDM apples had higher quinic acid content than GD at 7 and 87 DPA, which did not align with the trend seen in total organic acid content. This suggests that malic acid was the main factor influencing the acidity difference between the two varieties (Figure 1D). Tartaric (Figure 1E) and citric acid (Figure 1F) levels remained low throughout fruit development, likely having little to no significant impact on the fruit’s overall acidity.

### 3.2. Analysis of Differentially Expressed Genes (DEGs) Between GD and GDM Apples

**Figure 2:**
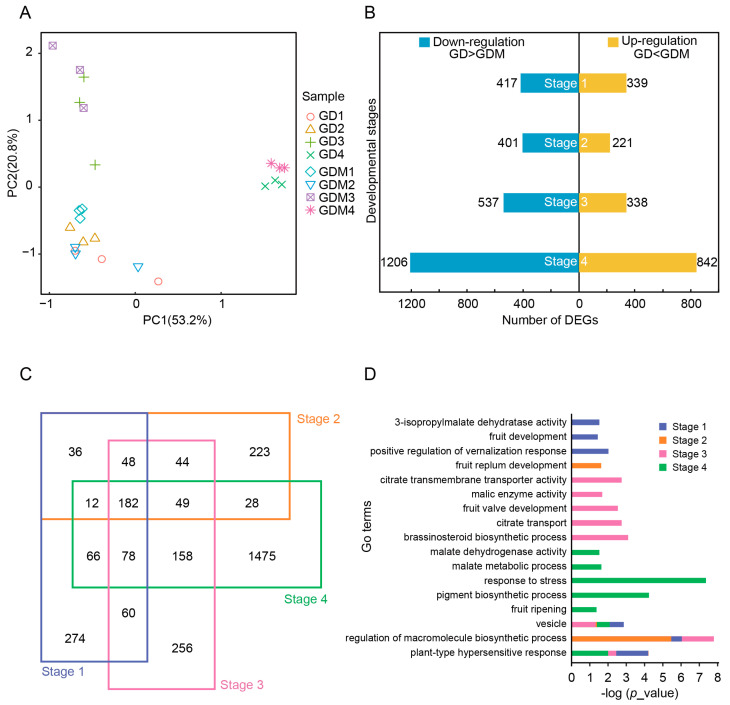
Transcriptome profiles of Golden Delicious (GD) and Golden Delicious Mutation (GDM) apples in different developmental stages. (**A**) Principal component analysis of all replicates of GD and GDM apples in four stages. (**B**) Bar plot showing number of differentially expressed genes (DEGs) in each stage in GD and GDM apples. (**C**) Venn diagram showing overlap of DEGs in different stages. (**D**) GO enrichment analysis of DEGs in different stages.

After determining and analyzing the content of various organic acids, we performed RNA-seq studies in four stages of development (27 d, 47 d, 67 d, and 147 d) of GD and GDM apple fruits. The first three stages represented the developmental phase, while the fourth stage corresponded to the maturation phase. Principal component analysis revealed that samples from the developmental stages of GD and GDM apples were closely clustered, whereas samples from the maturation stage were more similar to each other (Figure 2A).

We identified differentially expressed genes (DEGs) at each stage for both GD and GDM apples. The number of DEGs at the four stages was 756, 622, 875, and 2048 (Figure 2B), with the down-regulated genes consistently outnumbering the up-regulated genes. An analysis of the DEGs showed that the differences between GD and GDM apples were more pronounced in the maturation stage. We further analyzed DEGs across the different stages and found that many DEGs were shared between multiple stages. In the first, second, and third stages, 63.76%, 64.15%, and 70.74% of DEGs occurred in other stages, respectively (Figure 2C). This suggests that many differential genes persist throughout the development of both GD and GDM apples.

Additionally, we conducted a functional enrichment analysis of DEGs from different developmental stages. Given the presence of many common DEGs, several pathways were enriched across stages, including in terms of vacuole development, the regulation of macromolecule biosynthetic processes, and plant-type hypersensitive responses. Due to the significant maturation differences between GD and GDM apples, more biological processes were enriched in the maturation stage, including responses to stress, pigment biosynthetic processes, and fruit ripening. Furthermore, metabolic pathways related to organic acids, such as citrate transmembrane transporter activity and malate metabolic processes, were enriched in multiple stages (Figure 2D).

### 3.3. Dynamic Changes in Gene Expression During Fruit Ripening

**Figure 3:**
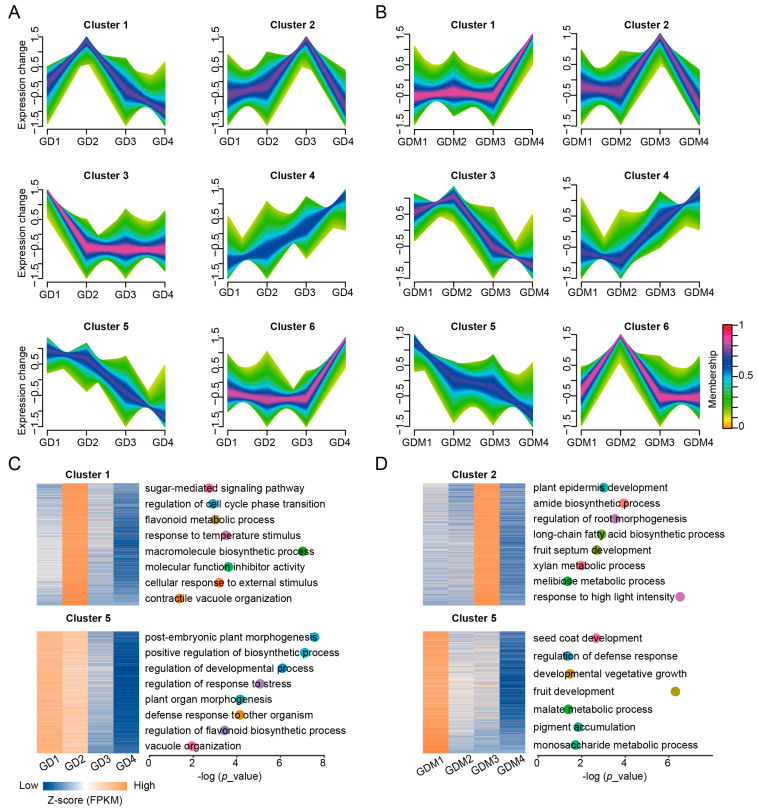
Clustering analysis of time series genes during fruit ripening. (**A**,**B**) Clustering patterns of expressed genes in GD (**A**) and GDM apples (**B**). (**C**,**D**) Enrichment analysis of key categories of genes in GD (**C**) and GDM apples (**D**). Different dots represent different GO terms, and their positions represent corresponding *p*_value.

Based on previous results, it is clear that DEGs affecting important biological functions exist in GD and GDM apples. To investigate the changes in gene expression during apple development, we performed a cluster analysis of genes expressed in the developmental stages of GD and GDM apples. These genes were clustered into six categories using the Mfuzz method (Figure 3A,B). Among these clusters, Cluster 6 for GD and Cluster 1 for GDM apples contained the most genes, implying that more genes were highly expressed in the maturation stage of apples, thus making them have more traits that caused differences among varieties. We also discovered that for GD, Clusters 1 and 5 were consistent with trends in their malic and quinic acid changes, respectively (Figure 3A), and for GDM apples, Clusters 2 and 5 were consistent with changes in quinic and malic acid content, respectively, during fruit development (Figure 3B). We hypothesized that there are genes in these important clusters that influence the changes in crucial organic acids in the fruit. Thus, we functionally enriched these categories and found that in addition to the key organic acid biosynthesis pathways, there was also an enrichment in the sugar-mediated signaling pathway, the flavonoid metabolic process, and contractile vacuole organization for GD (Figure 3C) and in fruit septum development, the amide biosynthetic process, and the developmental vegetative growth pathway for GDM apples (Figure 3D).

### 3.4. Co-Expression Network Analysis

**Figure 4:**
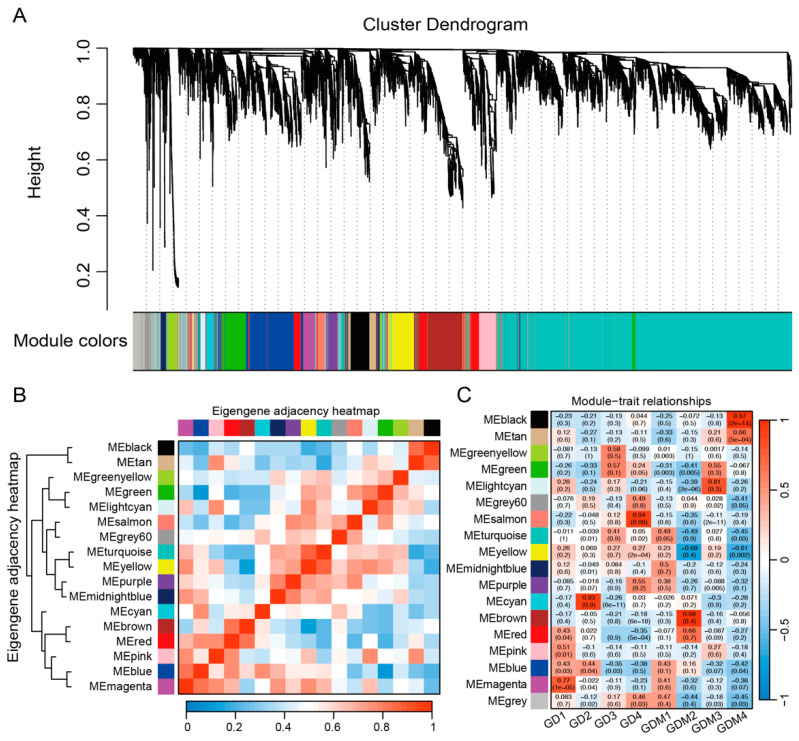
Identification modules related to fruit ripening shown via gene co-expression network. (**A**) Weighted gene co-expression network analysis identified 17 co-expression modules. (**B**) Clustering and correlation analysis between modules. (**C**) Relevance of modules and each developmental stage.

To further investigate the changes in the transcriptome of the two varieties of apples during fruit ripening, we integrated all the key genes from previous studies, including the DEGs in each developmental stage of GD and GDM apples, as well as key clusters, and performed weighted gene co-expression network analysis (WGCNA). Genes with similar expression patterns were grouped into 18 modules (Figure 4A). Among these modules, the turquoise and grey60 modules contained the highest and lowest number of genes, 3682 and 56, respectively. The clustering and correlation analyses of the modules revealed that the 18 modules were mainly classified into three categories: the first consisted of the black and tan modules; the second of cyan, brown, red, pink, blue, and magenta; and the third of the rest of the modules (Figure 4B). An analysis of the constructed modules and sample relationships revealed that the first category was significantly and positively correlated with the GDM ripening stage (fourth stage) and that most of the modules in the second category were correlated with the first stage (high-acidity stage) of the two varieties of apples (Figure 4C). In particular, two modules, magenta and blue, showed a positive correlation with the first stage of both varieties of apples, and we focused on analyzing these two modules. We found that the genes in these two modules influence malate dehydrogenase (decarboxylating) (NAD+) activity, transmembrane transport, and vacuolar membrane dynamics, in addition to being involved in important biological processes, such as the positive regulation of the carotenoid biosynthetic process, plant-type hypersensitive response, response to non-ionic osmotic stress, and the regulation of gene expression. These are as important as the biological pathways of organic acid synthesis and accumulation, thus suggesting that we identified important modules affecting the acidity quality of apples.

### 3.5. Patterns of Gene Regulation of Acidity in Apple Fruit

**Figure 5:**
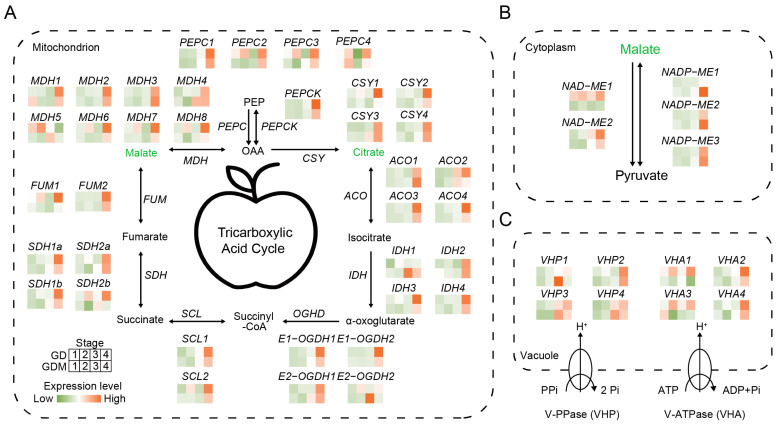
Patterns of intracellular acidity regulation and related gene expression. (**A**) Tricarboxylic acid cycle in mitochondria. (**B**) Patterns of acidity regulation in cytoplasm. (**C**) Proton pumps regulate acidity pattern of vacuole. qPEP: phosphoenolpyruvate; PEPC: phosphoenolpyruvate carboxylase; PEPCK: phosphoenolpyruvate carboxykinase; OAA: oxaloacetate; CSY: citrate synthase; ACO: Aconitase; IDH: Isocitrate Dehydrogenase; OGHD: Oxoglutarate dehydrogenase; SCL: Succinyl-CoA Synthetase; SDH: Succinate Dehydrogenase; FUM: Fumarase; MDH: malate dehydrogenase; ME: melic enzyme.

The organic acids accumulated in apples are primarily synthesized within the fruit itself, with different types of organic acids being synthesized in distinct parts of the fruit, leading to variations in overall acidity [37]. However, the primary storage site for organic acids is the vacuole. Studies have shown that the organic acid content at fruit maturity is determined by the balance between organic acid synthesis, vacuole storage, and translocation [38]. The two main organic acids, citric acid and malic acid, are primarily derived from the tricarboxylic acid (TCA) cycle in the mitochondria. In this study, we analyzed the expression patterns of key genes in the TCA cycle for the two apple varieties, GD and GDM apples. For *citrate synthase* (*CSY*), which is the key gene involved in citric acid synthesis, no significant differences in expression were observed during the developmental stages of GD and GDM apples, which is consistent with the measured citric acid content in both varieties. In contrast, for *malate dehydrogenase* (*MDH*), which is responsible for malic acid synthesis, the expression levels of several *MDH* copies were higher in GD compared to GDM apples during the developmental stage. Notably, the expression pattern of *MDH5* closely mirrored the trend in malic acid content in GD, suggesting that *MDHs*, particularly *MDH5*, play a key role in malic acid accumulation during the early developmental stages. This difference in *MDH* expression likely contributes to the observed variations in fruit acidity between GD and GDM apples (Figure 5A).

In addition to the mitochondria, the conversion of malate–pyruvate in the cytoplasm serves as another important regulatory mechanism for malate metabolism. Our analysis revealed that *NAD-malate enzyme* (*NAD-ME*) exhibited consistently higher expression levels in GD compared to GDM apples throughout fruit development. We hypothesize that this may be linked to the consistently higher malate content observed in GD relative to GDM apples. Furthermore, GD displayed a faster malate degradation rate compared to GDM apples, supporting the role of *NAD-ME1* in driving these differences (Figure 5B).

Although organic acids are primarily synthesized in different organelles, their accumulation predominantly occurs in the vacuole, making transport carriers critical for transmembrane transport. Previous studies in citrus fruits and other species have shown that this transmembrane process is largely mediated by two independent vacuole membrane-bound proton pumps: H^+^-ATPase (VHA) and H^+^-PPase (VHP) [39,40]. In this study, we characterized the expression of these two key genes in apples. As expected, the expression levels of multiple *VHA1* isoforms were higher in GD compared to GDM apples (Figure 5C). This suggests that more organic acids were transported into the vacuoles of GD during fruit development, thus contributing to the higher overall organic acid content in GD. This mechanism is a significant factor underlying the greater acidity observed in GD compared to GDM apples.

### 3.6. Functional Validation of Critical Genes

**Figure 6:**
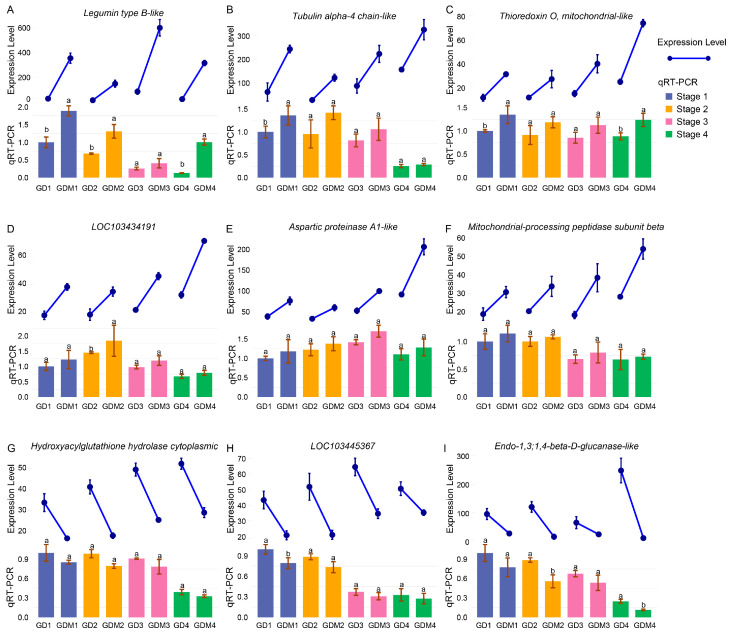
Transcriptome expression levels and qRT-PCR experiment results of 9 DEGs. Line and bar plots represent gene expression levels and qRT-PCR results, respectively. Groups 1, 2, 3, and 4 of GD and GDM apples represent four developmental stages of two apple varieties. (**A**–**I**) indicate 9 genes with gene IDs LOC103409144, LOC114823285, LOC103421679, LOC103434191, LOC114827605, LOC103442405, LOC103445367, LOC103438582, and LOC103424034. Groups labeled with the same letter (a, a) are not significantly different, while those with different letters (a, b) indicate statistically significant differences.

To validate the reliability of our transcriptome sequencing results, we selected nine differentially expressed genes (DEGs) that exhibited significant expression differences between the two varieties in each developmental stage for qRT-PCR analysis. The results demonstrated consistency between the qRT-PCR findings and the transcriptome data (Figure 6), thereby confirming the accuracy and reliability of our experimental outcomes.

## 4. Discussion

Golden Delicious (GD) is one of the world’s most popular apple varieties, celebrated for its unique taste and exceptional flavor. As a classic medium-ripening variety, it is widely cultivated in major apple-producing regions, including China, the United States, and Europe [41]. In the Sichuan Plateau region of China, we identified a variant of GD with several remarkable traits, such as a glossy fruit surface, minimal russeting, and excellent storage longevity. However, its most notable characteristic is its low acidity. This variant is particularly appealing to consumers who prefer low-acid apples, and its resistance to storage makes it ideal for large-scale cultivation due to an extended marketing period. To understand the basis of this apple’s low-acid quality, we conducted a detailed analysis of organic acid composition and content. The results revealed significant differences between the variant and standard GD apples. Notably, the malic acid content in the variant at maturity was only one-third of that in GD, making malic acid the primary determinant of this apple’s low-acid quality. Although quinic acid also constitutes a significant portion of the total organic acids during fruit development, the content differences between the two varieties were minimal. This suggests that quinic acid may contribute to the overall reduction in acidity during fruit development but does not play a decisive role in the variation in acidity between the two varieties. Usually, the organic acids in apples are dominated by malic acid, while quinic acid is more present in the bound state. The formation of quinic acid relies on the mangiferic acid pathway in plants and is further released during processing or microbial activities. Their functions cover metabolic regulation, health protection and industrial applications, reflecting the value of the diversity of plant secondary metabolites. Thus, the low acidity of this GD variant is primarily attributed to reduced malic acid levels, highlighting its potential as a unique and consumer-friendly apple variety.

In this study, we found that differences in the expression of MDH in the tricarboxylic acid (TCA) cycle may play a critical role in the variation in malic acid levels between the two apple varieties. This is consistent with previous research findings. Experiments conducted in low-altitude regions have confirmed that overexpression of MDHs increases malic acid content in both transgenic apples [42] and ripe apples [43]. This suggests that the mechanism of malic acid accumulation in apples is consistent at both high and low altitudes. The higher malic acid content in GD leads to elevated *NAD-ME1* expression, which in turn results in a faster rate of malic acid degradation. This is likewise consistent with the conversion of malic acid to pyruvic acid found in previous refrigerated apple studies [44]. This is the main process of malic acid synthesis and catabolism through TCA in apples. The proton pumps VHA and VHP are essential for providing the energy required for the transmembrane transport of organic acids [45]. Our results demonstrated that proton pump activity directly influences organic acid accumulation in apples, with VHA consistently playing a dominant role throughout fruit development. MdVHP1-2 enhances fruit quality by regulating sugar and acid levels, thus improving flavor in apples and other fruits [46]. Similar findings have been reported in citrus [47], grapes [48], and pears [49]. There are several important transcription factors, such as MdESE3, that play an important role in MdMa11, MdtDT, and MdMDH12 transcriptional regulation and malate accumulation [25]. Further studies on quinic acid, another significant organic acid in apples, are urgently needed to better understand its regulatory mechanisms. Research on apple organic acid regulation has already explored various aspects, including genetic variation [50], epigenetic modification [51], transcription factor regulation [25], and other contributing factors. However, for our unique and valuable varietal material, more comprehensive and in-depth studies are necessary. We plan to assemble a complete reference genome for new apple varieties in high-altitude regions and conduct epigenetic studies, analyzing DNA methylation and histone modification. These efforts aim to elucidate how epigenetic modifications mediate the effects of high-altitude environmental factors on key agronomic traits in apples.

## 5. Conclusions

This study highlights the low-acidity traits of the high-altitude variant of the Golden Delicious (GD) apple, in which reduced malic acid content is the primary factor contributing to its mild acidity. Differences in *malate dehydrogenase* (*MDH*) expression and proton pump activity (*VHA*) were key to this variation. While quinic acid also contributes to organic acid composition, it does not significantly impact acidity differences between the two varieties. These findings deepen our understanding of organic acid regulation in apples and provide a foundation for breeding fruit varieties with desirable sensory qualities. The molecular insights gained can be applied to the development of new apple varieties with tailored acidity levels, thus catering to consumer preferences. Further research is needed to explore quinic acid regulation and apply these insights to other fruits, advancing the breeding of high-quality, low-acid varieties for broader agricultural use.

## Data Availability

The data sets generated and/or analyzed during the current study are available from the corresponding author upon reasonable request.

## Appendix A

**Table A1:** Primers for qRT-PCR analysis of DEGs.

Gene Name	Primer Forward	Primer Reverse
Actin	AATGGAACTGGAATGGTCAAGGC	TGCCAGATCTTCTCCATGTCATCCCA
LOC103409144	ACAAGCTGGGTTAAGTGCGA	CAGGCCTGCAAAAATAATACCA
LOC114823285	GAACTTCTGTCGAAACAGCCG	AGCAGCATCTGCCATTCCTT
LOC103421679	GTGTCCTCAGACTCCCCAAG	TTTGCGCACTTTTTGTTGCAG
LOC103434191	CTGCAACAAAAAGTGCGCAAA	CGGTATGTATTGCGCGAAGC
LOC114827605	ACTCGACCAGTGTTGCTGAG	ACGACACAATACAAGGGGGA
LOC103442405	TGTTCACTGGCTGCTCCATT	CAGAAGGATGAGCGTTCGGT
LOC103445367	CAACATGCTGCAATAAGGCCA	CATGCATGCGGTTCAGTACG
LOC103438582	GCACGCATTCTATCGCCAAG	TCGACGGTCGGCATATGTTT
LOC103424034	TCAAAACAGAAGGCAGGTGGT	ACGGTCTAGAACAAGATCGGG

## References

[1] Ma B., Chen J., Zheng H., Fang T., Ogutu C., Li S., Han Y., Wu B. (2015). Comparative assessment of sugar and malic acid composition in cultivated and wild apples. Food Chem..

[2] Sytar O., Hajihashemi S. (2024). Specific Secondary Metabolites of Medicinal Plants and Their Role in Stress Adaptation. Plant Secondary Metabolites and Abiotic Stress.

[3] Mahmood T., Anwar F., Abbas M., Boyce M.C., Saari N. (2012). Compositional variation in sugars and organic acids at different maturity stages in selected small fruits from Pakistan. Int. J. Mol. Sci..

[4] Albertini M.-V., Carcouet E., Pailly O., Gambotti C., Luro F., Berti L. (2006). Changes in Organic Acids and Sugars during Early Stages of Development of Acidic and Acidless Citrus Fruit. J. Agric. Food Chem..

[5] Melgarejo P., Salazar D.M., Artés F. (2000). Organic acids and sugars composition of harvested pomegranate fruits. Eur. Food Res. Technol..

[6] Famiani F., Battistelli A., Moscatello S., Cruz-Castillo J.G., Walker R.P. (2015). The organic acids that are accumulated in the flesh of fruits: Occurrence, metabolism and factors affecting their contents-a review. Rev. Chapingo Ser. Hortic..

[7] Patocka J., Bhardwaj K., Klimova B., Nepovimova E., Wu Q., Landi M., Kuca K., Valis M., Wu W. (2020). Malus domestica: A review on nutritional features, chemical composition, traditional and medicinal value. Plants.

[8] Daccord N., Celton J.-M., Linsmith G., Becker C., Choisne N., Schijlen E., Van de Geest H., Bianco L., Micheletti D., Velasco R. (2017). High-quality de novo assembly of the apple genome and methylome dynamics of early fruit development. Nat. Genet..

[9] Harris S.A., Robinson J.P., Juniper B.E. (2002). Genetic clues to the origin of the apple. Trends Genet..

[10] Mabrok H.B., Mohamed D.A., Sytar O., Smetanska I. (2019). Biological evaluation of golden delicious apples exposure to UV lights in rats. Pak. J. Biol. Sci..

[11] Li W., Chu C., Zhang T., Sun H., Wang S., Liu Z., Wang Z., Li H., Li Y., Zhang X. (2025). Pan-genome analysis reveals the evolution and diversity of Malus. Nat. Genet..

[12] Lin Q., Chen J., Liu X., Wang B., Zhao Y., Liao L., Allan A.C., Sun C., Duan Y., Li X. (2023). A metabolic perspective of selection for fruit quality related to apple domestication and improvement. Genome Biol..

[13] Zhang Y., Fernie A.R. (2018). On the role of the tricarboxylic acid cycle in plant productivity. J. Integr. Plant Biol..

[14] Nunes-Nesi A., Araújo W.L., Obata T., Fernie A.R. (2013). Regulation of the mitochondrial tricarboxylic acid cycle. Curr. Opin. Plant Biol..

[15] Ma B., Yuan Y., Gao M., Xing L., Li C., Li M., Ma F. (2018). Genome-wide Identification, Classification, Molecular Evolution and Expression Analysis of Malate Dehydrogenases in Apple. Int. J. Mol. Sci..

[16] Zheng Y.-j., Yang Z.-q., Wei T.-t., Zhao H.-l. (2022). Response of tomato sugar and acid metabolism and fruit quality under different high temperature and relative humidity conditions. Phyton-Int. J. Exp. Bot..

[17] Moing A., Rothan C., Svanella L., Just D., Diakou P., Raymond P., Gaudillère J.P., Monet R. (2000). Role of phosphoenolpyruvate carboxylase in organic acid accumulation during peach fruit development. Physiol. Plant..

[18] Huang X.-Y., Wang C.-K., Zhao Y.-W., Sun C.-H., Hu D.-G. (2021). Mechanisms and regulation of organic acid accumulation in plant vacuoles. Hortic. Res..

[19] Shiratake K., Martinoia E. (2007). Transporters in fruit vacuoles. Plant Biotechnol..

[20] Yu H., Zhang C., Lu C., Wang Y., Ge C., Huang G., Wang H. (2024). The lemon genome and DNA methylome unveil epigenetic regulation of citric acid biosynthesis during fruit development. Hortic. Res..

[21] Huang Y., He J., Xu Y., Zheng W., Wang S., Chen P., Zeng B., Yang S., Jiang X., Liu Z. (2023). Pangenome analysis provides insight into the evolution of the orange subfamily and a key gene for citric acid accumulation in citrus fruits. Nat. Genet..

[22] Lu Z., Huang Y., Mao S., Wu F., Liu Y., Mao X., Adhikari P.B., Xu Y., Wang L., Zuo H. (2022). The high-quality genome of pummelo provides insights into the tissue-specific regulation of citric acid and anthocyanin during domestication. Hortic. Res..

[23] Zhang L., Ma B., Wang C., Chen X., Ruan Y.-L., Yuan Y., Ma F., Li M. (2022). MdWRKY126 modulates malate accumulation in apple fruit by regulating cytosolic malate dehydrogenase (MdMDH5). Plant Physiol..

[24] Gao M., Yang N., Shao Y., Shen T., Li W., Ma B., Wei X., Ruan Y.-L., Ma F., Li M. (2024). An insertion in the promoter of a malate dehydrogenase gene regulates malic acid content in apple fruit. Plant Physiol..

[25] Zheng L., Ma W., Liu P., Song S., Wang L., Yang W., Ren H., Wei X., Zhu L., Peng J. (2024). Transcriptional factor MdESE3 controls fruit acidity by activating genes regulating malic acid content in apple. Plant Physiol..

[26] Wang Y. (2024). China’s apple production reigns as world’s No. 1. China Farmers’ Daily.

[27] Nisperos-Carriedo M.O., Buslig B.S., Shaw P.E. (1992). Simultaneous detection of dehydroascorbic, ascorbic, and some organic acids in fruits and vegetables by HPLC. J. Agric. Food Chem..

[28] Chen S. (2023). Ultrafast one-pass FASTQ data preprocessing, quality control, and deduplication using fastp. Imeta.

[29] Kim D., Paggi J.M., Park C., Bennett C., Salzberg S.L. (2019). Graph-based genome alignment and genotyping with HISAT2 and HISAT-genotype. Nat. Biotechnol..

[30] Pertea M., Pertea G.M., Antonescu C.M., Chang T.-C., Mendell J.T., Salzberg S.L. (2015). StringTie enables improved reconstruction of a transcriptome from RNA-seq reads. Nat. Biotechnol..

[31] Ritchie M.E., Phipson B., Wu D., Hu Y., Law C.W., Shi W., Smyth G.K. (2015). limma powers differential expression analyses for RNA-sequencing and microarray studies. Nucleic Acids Res..

[32] Kumar L., Futschik M.E. (2007). Mfuzz: A software package for soft clustering of microarray data. Bioinformation.

[33] Langfelder P., Horvath S. (2008). WGCNA: An R package for weighted correlation network analysis. BMC Bioinform..

[34] Klopfenstein D., Zhang L., Pedersen B.S., Ramírez F., Warwick Vesztrocy A., Naldi A., Mungall C.J., Yunes J.M., Botvinnik O., Weigel M. (2018). GOATOOLS: A Python library for Gene Ontology analyses. Sci. Rep..

[35] Schmittgen T.D., Livak K.J. (2008). Analyzing real-time PCR data by the comparative CT method. Nat. Protoc..

[36] Walker R.P., Famiani F. (2018). Organic acids in fruits: Metabolism, functions and contents. Hortic. Rev..

[37] Zhang L.-H., Zhang A.-N., Xu Y., Zhu L.-C., Ma B.-Q., Li M.-J. (2024). Accumulation and regulation of malate in fruit cells. Fruit Res..

[38] Etienne A., Génard M., Lobit P., Mbeguié-A-Mbéguié D., Bugaud C. (2013). What controls fleshy fruit acidity? A review of malate and citrate accumulation in fruit cells. J. Exp. Bot..

[39] Guo L.-X., Shi C.-Y., Liu X., Ning D.-Y., Jing L.-F., Yang H., Liu Y.-Z. (2016). Citrate accumulation-related gene expression and/or enzyme activity analysis combined with metabolomics provide a novel insight for an orange mutant. Sci. Rep..

[40] Yang J., Zhang J., Niu X.-Q., Zheng X.-L., Chen X., Zheng G.-H., Wu J.-C. (2021). Comparative transcriptome analysis reveals key genes potentially related to organic acid and sugar accumulation in loquat. PLoS ONE.

[41] Volk G.M., Olmstead J.W., Finn C.E., Janick J. (2013). The ASHS outstanding fruit cultivar award: A 25-year retrospective. HortScience.

[42] Zhang L., Wang C., Jia R., Yang N., Jin L., Zhu L., Ma B., Yao Y.-X., Ma F., Li M. (2022). Malate metabolism mediated by the cytoplasmic malate dehydrogenase gene MdcyMDH affects sucrose synthesis in apple fruit. Hortic. Res..

[43] Gao M., Zhao H., Zheng L., Zhang L., Peng Y., Ma W., Tian R., Yuan Y., Ma F., Li M. (2022). Overexpression of apple Ma12, a mitochondrial pyrophosphatase pump gene, leads to malic acid accumulation and the upregulation of malate dehydrogenase in tomato and apple calli. Hortic. Res..

[44] Zhao J., Quan P., Liu H., Li L., Qi S., Zhang M., Zhang B., Li H., Zhao Y., Ma B. (2020). Transcriptomic and Metabolic Analyses Provide New Insights into the Apple Fruit Quality Decline during Long-Term Cold Storage. J. Agric. Food Chem..

[45] Schumacher K. (2014). pH in the plant endomembrane system—An import and export business. Curr. Opin. Plant Biol..

[46] Xiang Y., Huang X.-Y., Zhao Y.-W., Wang C.-K., Sun Q., Hu D.-G. (2024). Optimization of apple fruit flavor by MdVHP1-2 via modulation of soluble sugar and organic acid accumulation. Plant Physiol. Biochem..

[47] Brune A., Müller M., Taiz L., Gonzalez P., Etxeberria E. (2002). Vacuolar acidification in citrus fruit: Comparison between acid lime (*Citrus aurantifolia*) and sweet lime (*Citrus limmetioides*) juice cells. J. Am. Soc. Hortic. Sci..

[48] Terrier N., Sauvage F.-X., Ageorges A., Romieu C. (2001). Changes in acidity and in proton transport at the tonoplast of grape berries during development. Planta.

[49] Suzuki Y., Shiratake K., Yamaki S. (2000). Seasonal changes in the activities of vacuolar H+-pumps and their gene expression in the developing Japanese pear fruit. J. Jpn. Soc. Hortic. Sci..

[50] Xu K., Wang A., Brown S. (2012). Genetic characterization of the Ma locus with pH and titratable acidity in apple. Mol. Breed..

[51] Ma W., Li B., Zheng L., Peng Y., Tian R., Yuan Y., Zhu L., Su J., Ma F., Li M. (2021). Combined profiling of transcriptome and DNA methylome reveal genes involved in accumulation of soluble sugars and organic acid in apple fruits. Foods.

